# Comparison of the effect of traditional Chinese medicine injection combined with chemotherapy and chemotherapy alone on the prognosis, quality of life and immune function in patients with ovarian carcinoma: A protocol for systematic review and network meta-analysis

**DOI:** 10.1097/MD.0000000000027395

**Published:** 2021-10-15

**Authors:** Xingnong Xu, Li Zhu, Lin Long

**Affiliations:** a Guang Xi Jiu Sheng New Materials CO.,LTD, Wuzhou, Guangxi Province, China; b School of Politics and Public Administration, Guangxi University for Nationalities, Nanning, Guangxi Province, China; c ZhongHeng Group, Wuzhou, Guangxi Province, China.

**Keywords:** efficacy, immune function, meta-analysis, ovarian carcinoma, protocol, traditional Chinese medicine injection

## Abstract

**Background:**

The effectiveness of traditional Chinese medicine (TCM) in assisting the reduction of the toxic effects of chemotherapy and enhancing the efficacy of chemotherapy is gradually being recognized. Traditional Chinese medicine injection (TCMJ) has been widely used as a promising adjuvant drug in the treatment of ovarian carcinoma. However, the exact clinical efficacy and safety of TCMJ have not been well studied due to the wide variety. This study aims to conduct a network meta-analysis of randomized controlled trials (RCTs) about comparing the effect of TCMJ combined with chemotherapy and chemotherapy alone on the treatment of ovarian cancer, thus summarizing the effects of TCMJ on the prognosis, quality of life and immune function of ovarian carcinoma patients, and providing a reference for developing therapeutic regimens for ovarian carcinoma.

**Methods:**

Randomized controlled trials reporting the effect of TCMJ combined with chemotherapy and chemotherapy alone on the prognosis, quality of life and immune function in patients with ovarian carcinoma published before September 2021 will be screened out from online databases like PubMed, Web of Science, Scopus, Cochrane Library, Embase, China Scientific Journal Database, China National Knowledge Infrastructure, Chinese Biomedical Literature Database, and Wanfang Database. Subsequently, 2 researchers will be independently responsible for literature screening, data extraction and assessment of their quality. All data will be processed by R.4.1.0.

**Results:**

The results of this meta-analysis will be submitted to a peer-reviewed journal for publication.

**Conclusions:**

Evidence-based medicine supports the efficacy and safety of TCMJ combined with chemotherapy for the treatment of ovarian carcinoma, which is better than that of chemotherapy alone.

**Ethics and dissemination:**

Ethical approval was not required for this study. The systematic review will be published in a peer-reviewed journal, presented at conferences, and shared on social media platforms.

**OSF REGISTRATION NUMBER:**

DOI 10.17605/OSF.IO/P93VJ.

## 1 Introduction

Ovarian carcinoma is one of the most common malignant tumors in gynecology, which is the leading cause of death in gynecological tumors.^[1–3]^ Among them, epithelial ovarian carcinoma accounts for about 90% of ovarian carcinomas, and its mortality ranks the first place among gynecological tumors, with a 5-year survival of only 30% to 40%.^[4]^

More than 70% of patients with ovarian carcinoma are diagnosed in the advanced stage.^[5]^ A combination of surgery and chemotherapy-based therapy is preferred to them.^[6–8]^ However, the cure rate is low, and the recurrence rate is up to 70% even after systematic treatment.^[6]^ The TC (paclitaxel + carboplatin) and TP (paclitaxel + cisplatin) regimens recommended by the International Federation of Obstetrics and Gynecology and domestic gynecologic oncology centers are the first-line chemotherapy regimens for ovarian carcinoma, which have achieved good clinical outcomes.^[9,10]^ However, toxic events like myelosuppression, gastrointestinal reactions, and liver and kidney function impairment caused by chemotherapy seriously affect the therapeutic efficacy and quality of life.^[11–13]^ Despite significant advances made in surgical techniques and drug therapy in recent years, the 5-year survival of ovarian carcinoma does not exceed 30%.^[14]^ Therefore, developing combination regimens with a higher efficacy and lower toxicity t is of significance to improve the quality of life and prognosis of ovarian carcinoma patients.

In recent years, the effectiveness of traditional Chinese medicine (TCM) in assisting the reduction of the toxic effects of chemotherapy and enhancing the efficacy of chemotherapy has been gradually recognized.^[15–17]^ The combination of TCM and Western medicine in the treatment of ovarian carcinoma is now becoming widely applied in clinical practice.^[18,19]^ Traditional Chinese medicine injection (TCMJ) is a product of TCM combined with modern process technology, which is featured by rapid action, high bioavailability and good efficacy.^[20,21]^The exact efficacy of a variety of TCMJs combined with chemotherapy in the treatment of ovarian carcinoma has been validated.^[22–26]^ However, due to the wide variety of TCMJ, their specific role in the treatment process requires further explorations. It is still unclear how to select the optimal TCMJ in the combination chemotherapy of ovarian cancer. Therefore, this study aims to conduct a network meta-analysis of randomized controlled trials (RCTs) reporting the efficacy of TCMJ combined with chemotherapy and chemotherapy alone on the treatment of ovarian carcinoma, thus summarizing the effects of TCMJ on the prognosis, quality of life and immune function of ovarian carcinoma patients, and providing a reference for selecting the optimal treatment options.

## 2 Methods

### 2.1 Study registration

The protocol of this review was registered in OSF (OSF registration number: DOI 10.17605/OSF.IO/P93VJ). Besides, it was reported as per the statement guidelines of preferred reporting items for systematic reviews and meta-analyses protocol.^[27]^

### 2.2 Inclusion criteria for study selection

#### 2.2.1 Types of studies

All RCTs about the effect of TCMJ combined with chemotherapy and chemotherapy alone on prognosis, quality of life, and immune function in patients with ovarian carcinoma.

#### 2.2.2 Types of participants

Patients with ovarian carcinoma over 18 years old.

#### 2.2.3 Types of interventions

In the experimental group, ovarian carcinoma patients must be treated with TCMJ combined with chemotherapy. The control group received chemotherapy alone. There will be no restrictions about chemotherapy regimens, types of TCMJ, drug doses, frequencies, and follow-up durations.

#### 2.2.4 Types of outcome indexes

1) Primary outcomes: disease-free survival and overall survival.
2) Secondary outcome:
  Quality of life assessed by scores of various quality of life scales.
  Various immune factors, including CD3+, CD4+, CD8+, CD4+/CD8+ cell ratios, etc.
3) Adverse events: myelosuppression, liver, and kidney function inhibition, gastrointestinal adverse reactions, etc.

### 2.3 Exclusion criteria

1) Non-RCT.
2) Animal experiments, case reports, reviews, etc.
3) Repeatedly detected or published literature.
4) The absence of complete data or full text literature.

### 2.4 Data sources

All RCTs reporting the effect of TCMJ combined with chemotherapy compared with chemotherapy alone on the prognosis, quality of life, and immune function in patients with ovarian carcinoma published before September 2021 will be systematically searched in PubMed, Web of Science, Scopus, Cochrane Library, Embase, China Scientific Journal Database, China National Knowledge Infrastructure, Chinese Biomedical Literature Database, and Wanfang Database. References of included RCTs will be manually reviewed to avoid missing data.

### 2.5 Searching strategy

The details of PubMed search strategies are illustrated in Table 1, including all search terms; while similar search strategies were applied to other electronic databases.

**Table 1:** Search strategy in PubMed database.

Number	Search terms
#1	Ovarian meoplasms [MeSH]
#2	Ovarian carcinoma [title/abstract]
#3	Cancer of ovary [title/abstract]
#4	Ovarian cancer [title/abstract]
#5	Cancer of the ovary [title/abstract]
#6	Neoplasms, ovarian [title/abstract]
#7	Ovary cancer [title/abstract]
#8	Ovary neoplasms [title/abstract]
#9	Cancer, ovarian [title/abstract]
#10	Cancer, ovary [title/abstract]
#11	Cancers, ovarian [title/abstract]
#12	Cancers, ovary [title/abstract]
#13	Neoplasm, ovarian [title/abstract]
#14	Neoplasm, ovary [title/abstract]
#15	Neoplasms, ovary [title/abstract]
#16	Ovarian cancers [title/abstract]
#17	Ovarian neoplasm [title/abstract]
#18	Ovary cancers [title/abstract]
#19	Ovary neoplasm [title/abstract]
#20	OR/1–19
#21	Chinese herbal injections [title/abstract]
#22	Traditional Chinese medicine injections [title/abstract]
#23	Traditional Chinese medicine [title/abstract]
#24	OR/21-23
#25	Randomized controlled trial [MeSH]
#26	Controlled trial [title/abstract]
#27	Random∗ [title/abstract]
#28	Controlled clinical trial [title/abstract]
#29	Clinical trial [title/abstract]
#30	OR/25–29
#31	#20 and #24 and #30

### 2.6 Data collection and analysis

#### 2.6.1 Literature screening and data extraction

**Figure 1:**
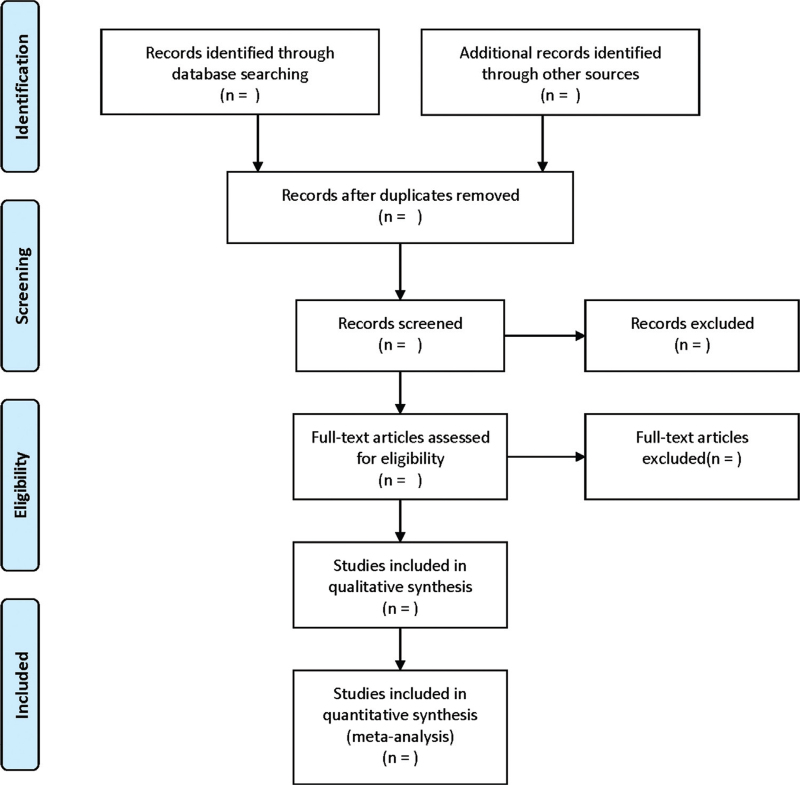
Flow diagram of study selection process.

According to the inclusion and exclusion criteria, 2 researchers independently completed the literature screening. By reading the full text, the data were extracted, and the final results were cross-checked. If there is any inconsistent opinion, it would be further negotiated and arbitrated with a third researcher. The extraction contents contain such aspects as the name of the first author, year of publication, country, gender, age, sample size, duration of follow-up, treatment, and dosage. The screening flow chart of this study is presented in Figure 1.

#### 2.6.2 Assessment of risk of bias

Two evaluators independently evaluated the quality of the included RCTs with the Cochrane Risk Assessment Manual.^[28]^ The evaluation results were classified into the high-risk, low-risk, and unclear categories.

#### 2.6.3 Measures of treatment effect

For dichotomous outcomes, risk ratio will be used in the meta-analysis. All of these data will be summarized with a 95% confidence interval (CI). Continuous variable data were represented by standardized mean difference and 95%CI. Survival data were expressed as hazard ratios and 95% CI.

#### 2.6.4 Management of missing data

If any data is absent, the original data will be requested by email. If there is a failure in the data request, such data would be excluded from the study.

#### 2.6.5 Assessment of heterogeneity and data synthesis

R.4.1.0 software was adopted to call the “gemtc” and “rjags” packages for statistical analysis. Direct and indirect comparisons between different drug interventions were presented by plotting a mesh relationship diagram. Based on the fixed effects model under Markov chain Monte Carlo consistency model, 4 chains were adopted for simulation, with the number of iterations set to 50,000, the number of annealing set to 10,000, and the step size set to 1. The first 10,000 were employed to eliminate the impacts of original values, and the last 40,000 were employed for sampling. Besides, the forest plot of each outcome indicator and rank probability ranking graph were plotted. Heterogeneity test: Q test was performed to qualitatively determine inter-study heterogeneity. If *P* ≥ .1, there is no inter-study heterogeneity; while if *P* < .1, there is inter-study heterogeneity. Meanwhile, I^2^ value was adopted to quantitatively evaluate the inter-study heterogeneity. If I^2^ ≤ 50%, it would be considered as good heterogeneity, and the fixed effects model would be adopted. If I^2^ > 50%, it would be considered as significant heterogeneity, and the source of heterogeneity would be explored through subgroup analysis or sensitivity analysis. If there is no obvious clinical or methodological heterogeneity, it would be considered as statistical heterogeneity, and the random effects model would be adopted for analysis. If there is significant clinical heterogeneity between both groups, the descriptive analysis would be conducted, while subgroup analysis is not required.

#### 2.6.6 Assessment of reporting biases

Funnel plot will be performed to analyze the existence of publication bias if 10 or more literatures are included in this meta-analysis.^[29]^

#### 2.6.7 Subgroup analysis

Subgroup analysis will be conducted based on types of chemotherapy regimen, types and courses of TCMJ.

#### 2.6.8 Sensitivity analysis

Through exploring the large weight of elimination effects, the sensitivity analysis was performed to test the stability of the meta-analysis results.

#### 2.6.9 Grading the quality of evidence

The Grading of Recommendations Assessment, Development, and Evaluation was adopted to evaluate the quality of evidence from the following 5 aspects: risk of bias, indirectness, inconsistency, imprecision, and publication bias.^[30]^

#### 2.6.10 Ethics and dissemination

The contents of this article do not involve moral approval or ethical review and would be presented in print or at relevant conferences.

## 3 Discussion

Currently, platinum-based combination chemotherapy is the first-line chemotherapy regimen for ovarian carcinoma. About 70% of advanced patients with ovarian carcinoma fail from chemotherapy due to recurrence, drug resistance, and intolerance to severe adverse reactions.^[31]^ In recent years, Chinese medical professions have combined chemotherapeutic drugs with TCMJ to increase the efficiency and reduce toxicity of chemotherapy.^[32]^ However, the specific function, efficacy and safety of TCMJ combined with chemotherapy in the treatment of ovarian carcinoma have not been analyzed in RCTs. Network meta-analysis can quantify and analyze different interventions for treating the same disease and rank each intervention to yield the best intervention. In this study, the effect of TCMJ on the prognosis, quality of life and immune function of ovarian carcinoma patients will be summarized and ranked using a network meta-analysis, which will provide a reference for selecting optimal TCMJs.

## Author contributions

**Conceptualization:** Xingnong Xu.

**Data curation:** Li Zhu.

**Formal analysis:** Xingnong Xu.

**Funding acquisition:** Xingnong Xu.

**Investigation:** Li Zhu.

**Methodology:** Li Zhu.

**Project administration:** Xingnong Xu.

**Resources:** Lin Long, Li Zhu.

**Software:** Lin Long.

**Supervision:** Xingnong Xu.

**Validation:** Lin Long.

**Visualization:** Lin Long.

**Writing – original draft:** Xingnong Xu.

**Writing – review & editing:** Xingnong Xu.
